# Plasma microrna quantification protocol

**DOI:** 10.20517/2574-1209.2023.69

**Published:** 2023-11-15

**Authors:** Sophie Maiocchi, Elizabeth N. Collins, Andrew R. Peterson, Kyle C. Alexander, Dalton J. McGlamery, Noah A. Cassidy, John S. Ikonomidis, Adam W. Akerman

**Affiliations:** 1 Department of Cell Biology and Physiology, University of North Carolina at Chapel Hill, Chapel Hill, NC 27599-7545, USA.; 2 Department of Surgery, Division of Cardiothoracic Surgery, University of North Carolina at Chapel Hill, Chapel Hill, NC 27599-7065, USA.; 3 University of North Carolina School of Medicine, Chapel Hill, NC 27599, USA.

**Keywords:** MicroRNA, plasma, quantification, aortic aneurysm, ddPCR

## Abstract

MicroRNAs (miRNAs) are small non-coding RNA molecules that regulate translation and are involved in many pathological processes. They have emerged as promising biomarkers for diagnosis of conditions such as aortic aneurysm disease. Quantifying miRNAs in plasma is uniquely challenging because of the lack of standardized reproducible protocols. To facilitate the independent verification of conclusions, it is necessary to provide a thorough disclosure of all pertinent experimental details. In this technical note, we present a comprehensive protocol for quantifying plasma miRNAs using droplet digital PCR. We detail the entire workflow, including blood collection, plasma processing, cryo-storage, miRNA isolation, reverse transcription, droplet generation, PCR amplification, fluorescence reading, and data analysis. We offer comprehensive guidance regarding optimization, assay conditions, expected results, and insight into the troubleshooting of common issues. The stepwise normalization and detailed methodological guide enhance reproducibility. Moreover, multiple portions of this protocol may be automated. The data provided in this technical note is demonstrative of the values typically obtained when following its steps. To facilitate standardization in data reporting, we include a table of expected aortic aneurysm-related miRNA levels in healthy human plasma. This versatile protocol can be easily adapted to quantify most circulating miRNAs in plasma, making it a valuable resource for diagnostic development.

## INTRODUCTION

MicroRNAs (miRNAs) are small non-coding RNA molecules that play a crucial role in regulating translation. They are secreted by most cell types and involved in a variety of physiological and pathological processes. Moreover, recent studies demonstrate that miRNAs are promising potential biomarkers for disease diagnosis, prognosis, and therapy response^[1,2]^. Circulating miRNAs - that is, those found in plasma - are of particular interest because they are relatively stable and easily accessible. Moreover, we have established that patients with thoracic aortic aneurysms have a unique plasma miRNA profile, which has demonstrated exceptional sensitivity and specificity as a diagnostic tool^[3]^.

However, quantifying miRNAs in plasma is challenging for several reasons, chief among them being a lack of reproducible, standardized protocols. Because there are many ways to perform each step of this protocol, results are often not comparable between studies. The foundation of this protocol is the standardization of blood collection and storage, miRNA isolation and normalization, and multiplexed target quantification. As such, the use of this protocol will facilitate multi-study comparison and allow systematic review and meta-analyses.

Digital droplet PCR (ddPCR) has immense potential and is a viable tool with which to overcome the many challenges intrinsic to measuring miRNAs in plasma. Herein, we present a validated method for the absolute quantification of circulating, cell-free miRNA utilizing miRNA-specific primers and DNA binding dyes measured by ddPCR. We employ two normalization steps: firstly, the input amount of miRNA is standardized, and secondly, we utilize an exogenous miRNA (miR-39) spike-in to normalize the cDNA generation step. ddPCR relies on the dilution and partitioning of a sample into thousands of individual droplets. Some will contain the target of interest, while others do not. PCR amplifies fluorescence intensity only in the droplets containing the targets of interest. A droplet reader then counts each droplet to determine the number of positive and negative values for each miRNA. This process allows for the absolute quantification of the copy number of a target miRNA. Previous studies have demonstrated that ddPCR quantification of miRNA results in much greater precision of values obtained compared to real-time PCR, in addition to absolute quantification^[4]^. Several previous studies have also applied ddPCR for the quantification of circulating cell-free miRNA^[4–8]^.

This technical note delineates the workflow for the quantification of miRNAs in its entirety: we begin with sample collection/preparation, reverse transcription, droplet generation, PCR amplification, and finish with data analysis. We have also provided insights and guidance regarding optimization, assay conditions, and the troubleshooting of common issues. Stepwise normalization and detailed workflow allow for reproducibility. It can easily be adapted to quantify most circulating miRNAs in plasma, making it a valuable resource for diagnostic development.

## PROTOCOL

### 1. Preparation of miR-39 exogenous spike-in [Figure 1].

A stepwise protocol is available in the [Supplementary File 1].

#### Resuspension and serial dilution of miR-39 exogenous spike-in

**Figure 1.**
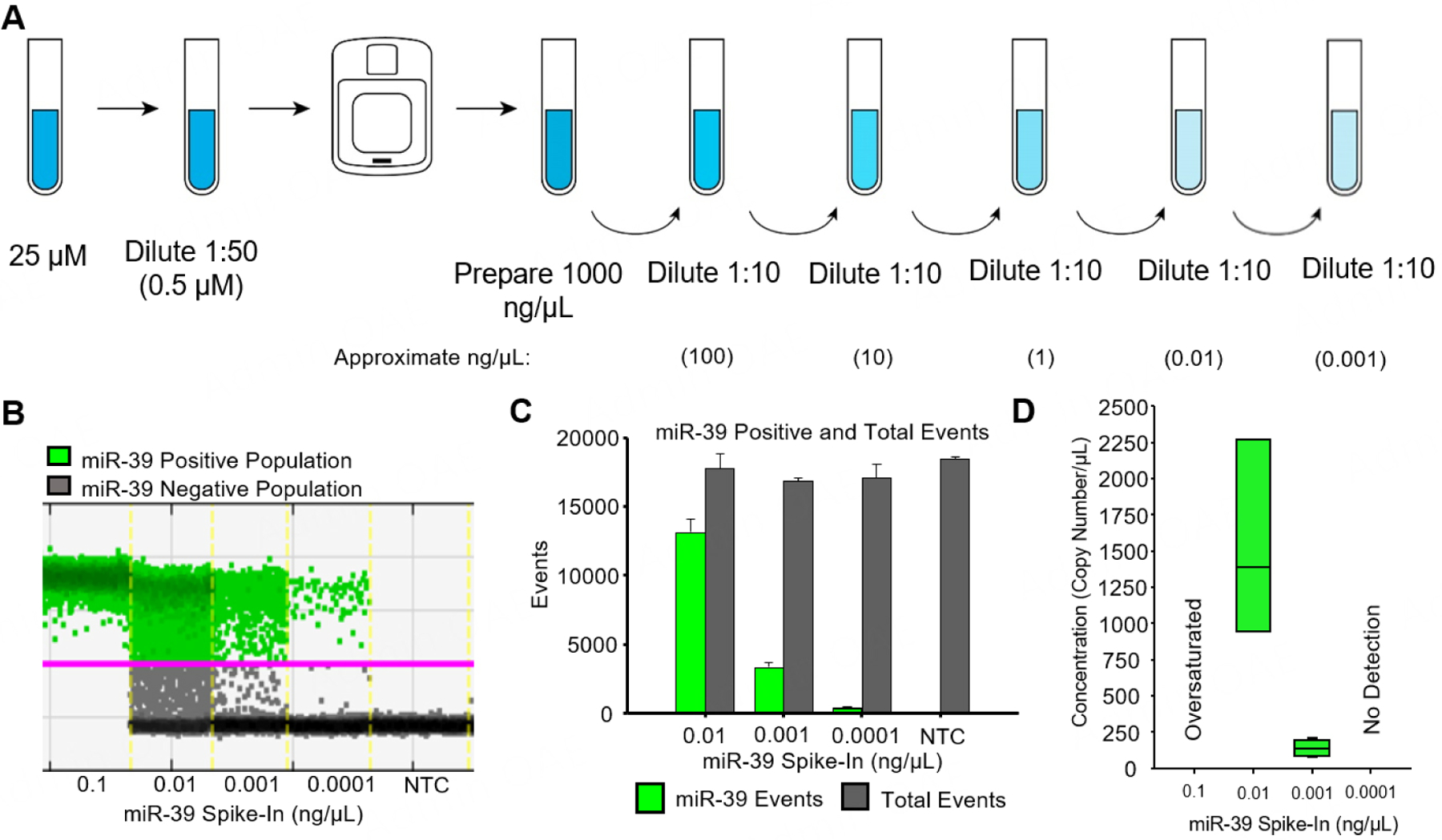
Preparation and Serial Dilution of Exogenous Spike-in Control. (A) Serial dilution instructions for creating a 25 μM stock solution of cel-miR-39–3p (miR-39) spike-in. First, a 0.5 μM solution of miR-39 spike-in is prepared by performing a 1:50 dilution in water. The actual stock concentration (in ng/μL) is then measured by a Qubit Fluorometer and microRNA Concentration Kit. The concentration of the stock solution is adjusted to 1,000 ng/μL. Serial dilutions of the miR-39 spike-in are prepared in water as shown. All dilutions are prepared by mixing 1 part of miR-39 spike-in solution with 9 parts water, vortexing (30 s) and resting on ice (30 s) 5 times before subsequent dilution. (B) Representative 1-Demensional analysis of 01–0.0001 ng/μL and the respective No Template Control (NTC). The green dots represent VIC positive (miR-39) particles, while the grey dots represent droplets with no fluorescence. (C) Ratio of miR-39 positive to total events. Results represent triplicate measurements, ±standard error of the mean (SEM). (D) Measured concentrations of miR-39 (copy number/μL), where the solid line is the median, and the bar represents the interquartile range.

The first step in this workflow is to prepare the exogenous spike-in, cel-miR-39-3p. Normally, miR-39 mimic oligonucleotides are supplied in lyophilized form following standard purification at either 5 or 20 nmol (mirVana^®^ miRNA mimic Assay ID: MC10956; miRbase v22.1 Accession Number: MI0000010). The following instructions and corresponding Figure 1A detail the preparation of the spike-in from a tube containing 5 nmol lyophilized. To prepare a 25 μM stock solution of miR-39 spike-in, begin by centrifuging the original tube (500× g for 1 min at room temperature), then resuspend in 200 μL of nuclease-free, molecular-biology-grade water. *Note: Diethylpyrocarbonate (DPEC)-treated water is not appropriate for use in this protocol due to the potential presence of residual DPEC, which can interfere with PCR components and cause nucleic acid damage. Hereafter, any reference to water pre-supposes nuclease-free, molecular-biology-grade water*. Thoroughly vortex the stock solution to achieve a homogenous mixture. Next, prepare a 0.5 μM working solution by performing a 1:50 dilution in water. Mix the dilution thoroughly by vortexing for 30 s. Using 1 to 10 μL of the prepared 0.5 μM working solution, measure the actual concentration in ng/μL using a Qubit Fluorometer and a miRNA Concentration Kit according to the instrument’s standard procedure for miRNA quantification (Catalogue #: Q32881).

After determining the actual concentration in ng/μL, prepare a 1,000 ng/μL dilution based on the actual concentration determined by the Qubit Assay. Next, prepare serial (1:10) dilutions from the 1,000 ng/μL stock. Vortex for 30 s, and rest on ice for an additional 30 s. Repeat this process 3 times before transferring the appropriate volume and performing a 1:10 dilution series resulting in concentrations of: 100, 10, 1, 0.1, 0.01, and 0.001 ng/μL.

#### cDNA generation of miR-39 exogenous spike-in

Next, measure the copy number of the prepared spike-in dilutions. For this, cDNA generation and ddPCR must be performed. To perform cDNA generation, follow the instruction manual for the TaqMan miRNA Reverse Transcription Kit from Applied Biosystems (Catalogue # 4366597). The following is a summary that includes precise numerical values for optimized volumes. Begin by preparing a miR-39 cDNA master mix using the following components per reaction: 0.15 μL of dNTP Mix, 0.19 μL of RNase Inhibitor, 1.5 μL of 10X RT Buffer, 1 μL of Multiscribe Reverse Transcriptase, 0.75 μL of miR-39 RT Primers (20×), and 10.41 μL of water, making a total volume of 14 μL. Add 14 μL of the master mix to all tubes. Add 1 μL of each miR-39 template to their respective tubes. Next, prepare the Negative Template Control (NTC) cDNA reaction by substituting the miR-39 template with 1 μL of water. *Please note: make an additional 10% excess volume for all reaction mixes to account for lost volume during routine pipetting*.

Place all tubes in a thermocycler. Set the thermocycler to the following conditions: 5 min at 4 °C, 30 min at 16 °C, 30 min at 42 °C, 5 min at 85 °C, and hold at 4 °C forever; lid temperature of 105 °C. Store the cDNA at −20 °C; ddPCR analysis must be performed within one year of cryostorage.

#### ddPCR analysis of miR-39 exogenous spike-in

Quantify the copy number of each miR-39 cDNA dilution. First, prepare a 20× concentration of miR-39 TaqMan probe (Applied Biosystems, catalog number: 4427975, Assay ID: 000200 VIC-MGB labeled). *Note: If necessary, dilute any probes supplied to a 20X concentration in water*. To perform ddPCR analysis, follow the instruction manuals for the TaqMan miRNA Assay from Applied Biosystems and the Droplet Digital PCR Applications Guide from Bio-Rad. The following is a summary that includes precise numerical values for optimized volumes. Begin by preparing a miR-39 ddPCR master mix using the following components per reaction: 12.5 μL of ddPCR Supermix (no dUTP); 1.25 μL of miR-39 VIC-labeled Probe (20×); 6.25 μL of water. The resulting total volume should be 20 μL. After vortexing the master mix, spin it down using a tabletop centrifuge, and carefully pipette 20 μL into the corresponding reactions. Spin down the cDNA using the tabletop centrifuge and add 5 μL of the appropriate cDNA to each reaction, resulting in a final volume of 25 μL. Prepare a Negative Control (NC) ddPCR “blank” reaction by adding 5 μL of water in place of the cDNA for a total volume of 25 μL.

Next, partition the reactions into droplets using the Bio-Rad Droplet generator. Place the DG8 droplet generator cassette (Bio-Rad DG8 Cartridges Catalog number: 1864008) into the droplet generator case (Bio-Rad Catalog number: 1863051), ensuring full closure and correct positioning of the plate. Using a multi-channel pipette, gently mix and transfer 20 μL of the samples to the row labeled “sample” on the droplet generator cassette. Subsequently, pipette 70 μL of droplet generator oil from a sterile trough into the row labeled “oil” on the droplet generator cassette. Exercise caution to not introduce bubbles. Securely place the rubber gasket on top of the droplet generator plate and generate droplets.

Once the droplet generator process is complete, the row marked as “droplets” on the plate should appear cloudy. Carefully transfer 40 μL of the droplets by gently withdrawing the pipette tip from the bottom of the well at a 35-degree angle, slowly withdrawing over a period of 5 s. Transfer the droplets into a deep, 96-well plate (Bio-Rad Catalog number: 12001925) by gently touching the pipette tip to the side of the well at the halfway point and dispense slowly, taking care not to introduce bubbles or rupture any droplets. Using a PX1 PCR Plate Sealer (Bio-Rad), seal the 96-deep well plate with pierceable foil (Bio-Rad Catalog number: 1814040) following the manufacturer’s instructions (180 °C for 5 s). Verify air-tight seals over each well opening by checking for complete circles impressed into the foil.

Perform PCR by placing the sealed plate into a deep-well 96-well thermocycler. Set the thermocycler conditions: 95 °C for 10 min (ramp 2 °C/s); 94 °C for 30 s (ramp 2 °C/s); 60 °C for 1 min (ramp 2 °C/s); repeat at step 2, 39 times; 98 °C for 10 min (ramp 2 °C/s); hold at 4 °C indefinitely; lid temperature: 105 °C.

Following PCR, remove the plate from the thermocycler and let it rest at room temperature for 5 min. Do not freeze. Perform droplet reading within 24 h following the instructions for the Bio-Rad QX200 Droplet Reader using the official Bio-Rad QX200 Droplet Reader Instruction Manual.

After completing the steps in the instruction manual, record the actual copy number of every lot. When selecting a spike-in dilution, it is important to choose a robust dilution that does not oversaturate or fall outside of the linear dynamic range of the targets of interest. For the miRNA targets presented in this technical note, we recommend choosing a spike-in dilution that is approximately 500–1,000 copies per μL.

Figure 1B is a representative one-dimensional analysis of 0.1–0.0001 ng/μL miR-39 dilutions and the respective NTC. The green dots represent VIC positive (miR-39) particles, while the grey dots represent droplets with no fluorescence. To demonstrate that all analyses are performed within the linear dynamic range of fluorescence detection, Figure 1C represents the ratio of miR-39 positive to negative particles. Measured concentrations of miR-39, in copy per μL, are reported in triplicate in Figure 1D. The average of these triplicates is used to record copy numbers for each lot of miR-39 cDNA spike-in prepared.

From the dilution selected, prepare small-volume aliquots. Vortex the large volumes for 30 s, followed by a 30-s rest on ice. Repeat this process 3 times, then aliquot into microcentrifuge tubes, and store at −80 °C until further use. *Note: once an aliquot has undergone 3 freeze-thaw cycles, it should no longer be used*. When ready, thaw the aliquots on ice or at 4 °C.

### 2. Blood collection, plasma isolation, and long-term storage [Figure 2A]:

To collect and isolate plasma from human blood, perform the following steps. First, a trained health care professional collects 5 mL of peripheral venous blood using an 18-gauge angiocatheter into a prelabeled 13 mm × 75 mm, plastic, BD Vacutainer^®^ EDTA-coated tube (K2 EDTA 7.2 mg, catalog number: 367861). *Note: EDTA is the recommended anticoagulant for miRNA quantification using this protocol*. Immediately after collection, invert the tube several times until it is thoroughly mixed and store it on ice for no more than 2 h. If not stored on ice, centrifuge the collected blood sample immediately at 2,500× g for 15 min at room temperature. This process separates the plasma from the rest of the whole blood components. Carefully collect the topmost layer (the plasma) of the supernatant and transfer it to a sterile, nuclease-free 15 mL conical tube. *Note: It is crucial to avoid disrupting the middle layer, known as the buffy coat. To do so, leave approximately 1 cm of plasma above it*.

Centrifuge the transferred plasma at 10,000× g for 15 min at room temperature, further purifying it and removing any unwanted cellular debris. Transfer only the topmost soluble volume into a new 15 mL conical tube. *Note: Leave the bottom ~100 μL of plasma in the tube to avoid any remaining impurities*. Next, aliquot desired volumes into appropriately labeled, freezer-safe, nuclease-free microcentrifuge tubes. *Note: a minimum aliquot volume of 250 μL is recommended for this protocol*. Snap freeze plasma fractionations in liquid nitrogen or a slurry of dry ice and isopropanol and transfer to −80 °C for long-term cryo-preservation. This ensures the integrity of miRNAs in the collected plasma samples for later use.

While performing the above steps, it is important to monitor for hemolysis. Hemolysis is the rupture of red blood cells and the release of hemoglobin. Hemolyzed samples will appear pink or red in color and must be excluded from analysis.

Plasma may be stored at −80 °C for up to two years. Once a single aliquot of plasma is thawed, you must perform all operations through the cDNA generation process. After the cDNA generation process has been completed, you can preserve specimens long-term (for up to one year) in cryopreservation.

### 3. Small RNA isolation from plasma [Figure 2B]:

A stepwise protocol is available in the supplement [Supplementary File 2].

We utilized the Qiagen miRNeasy Serum/Plasma Advanced Kit (Catalogue # 217204) as it has been reported to be a robust and reproducible method for miRNA purification from plasma^[9]^. We confirmed that in our hands, we found that we consistently isolated sufficient amounts of miRNA. We follow all instructions from the manufacturer, which can be found in the user manual. The following is a summary that includes precise numerical values for optimized reaction volumes and conditions.

MiRNA may be extracted from plasma by performing the following: First, retrieve the aliquoted 250 μL plasma samples from the −80 °C freezer and thaw them either on ice or at 4 °C. Thawing takes approximately 1 h. Vortex, then transfer 240 μL of plasma into a 2 mL microcentrifuge tube and centrifuge at 1,000× g for 10 min at 4 °C. Then, pipette 200 μL of the plasma supernatant to a new 2 mL microcentrifuge tube and add 60 μL of buffer RPL, vortex for 5 s, and then incubate for 3 min at room temperature. Following incubation, add 20 μL of buffer RPP to each tube, vortex for 30 s, and incubate again for 3 min at room temperature.

Centrifuge at 12,000× g for 3 min at room temperature. This should result in a clear and colorless supernatant. Transfer the supernatant to a new microcentrifuge tube, add an equal volume of 100% isopropanol, and vortex for 5 s. Transfer the full volume (approximately 500 μL) into a miRNeasy UCP MinElute column nested inside a clean, 1.5 mL microcentrifuge tube and spin at 8,000× g for 15 s. Discard the flow-through. *Note: To perform the wash steps, add subsequent volumes of reagents directly into the center of the column*. Add 700 μL of buffer RWT, centrifuge for 15 s at 8,000× g and discard the flow-through. Next, add 500 μL of buffer RPE, centrifuge for 15 s, and discard flow-through. Add an additional 500 μL of 80% ethanol and centrifuge for 2 min. Discard the flow-through. Transfer the miRNeasy UCP MinElute spin column to a new collection tube and leave the lid open. Centrifuge at full speed for 5 min; discard any remaining flow-through and the collection tube. Place the miRNeasy UCP MinElute spin column into a new 1.5 mL collection tube and add 20 μL of water directly to the center of the spin column membrane. Incubate for 10 min at room temperature. Finally, centrifuge for 1 min at full speed to elute the isolated miRNA. *Note: 18 μL of total miRNA is normally recovered following elution*. Proceed to small RNA quantification and concentration normalization immediately.

### 4. Small RNA quantification and concentration normalization [Figure 2C]:

Using 1 μL of the eluted miRNA, quantify concentration in ng/μL using the Qubit miRNA Assay and following the manufacturer’s instructions. After the concentration is measured, dilute the miRNA with RNase-free water such that the miRNA concentration will be 122.55 ng/μL.

**Figure 3.**
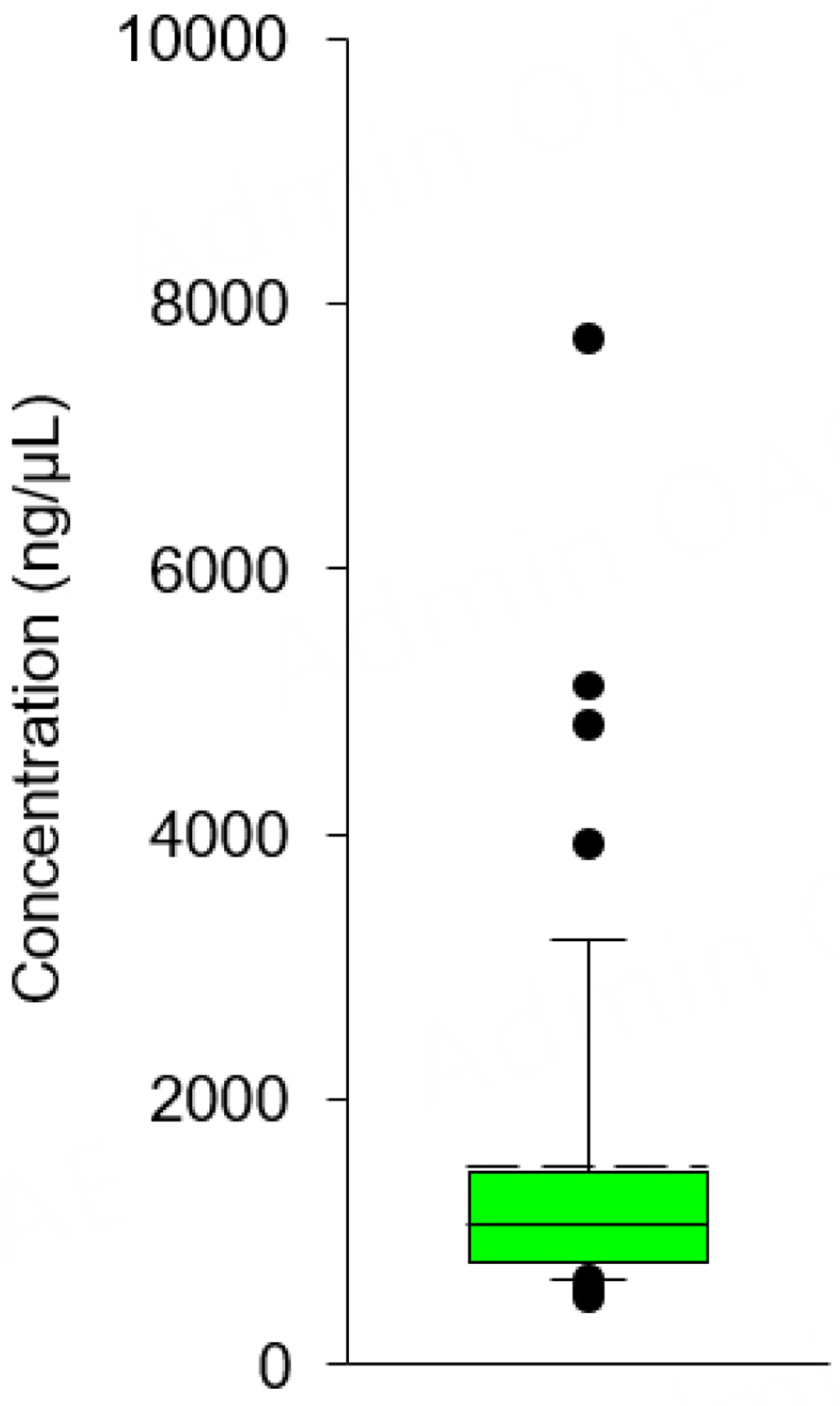
Expected range of microRNA concentration following isolation. microRNA is isolated from plasma with the Qiagen miRNeasy Serum/Plasma Advanced Kit, and the concentration of microRNA is quantified by the Qubit microRNA assay. Results represent 47 independent biological samples where the dotted line is the mean, and the solid line is the median, and the bar represents the interquartile range.

Figure 3 represents expected miRNA concentrations following quantification using the Qubit miRNA assay. Median values obtained from 47 different healthy human subjects were 1,062 ± 202.01 ng/μL. The standard deviation was 1,384.90 ng/μL. The 25th and 75th interquartile ranges were 760 and 1,458 ng/μL, respectively, with a coefficient of variation of 13.54. *Note: If measured values fall outside the range reported in* Figure 3*, results likely indicate degradation or contamination. Compromised samples with concentrations outside these ranges must be excluded from subsequent analysis*.

Once you have completed the above steps, roughly 18,054 ng of total miRNA remains for analysis. This allows for quantification of approximately 9 miRNA targets per 200 μL of plasma. Proceed to spike-in addition and miRNA-specific cDNA generation immediately.

### 5. Spike-in addition and miRNA-specific cDNA generation [Figure 2D]:

Once total miRNA has been corrected to a concentration of 122.55 ng/μL proceed to cDNA generation. For cDNA generation, a miR-39 counterpart is prepared separately for each miRNA target under investigation. Target-specific and miR-39-specific cDNA generation will be performed separately to avoid cross-reactivity. Supplementary Figure 1 is an example of results when cDNA generation is performed in a single tube. The skewed negative population resulting from the cross-reactivity impedes the ability to accurately set a threshold and acquire reproducible data.

miRNA target specific cDNAs will be generated using the TaqMan miRNA Reverse Transcription Kit (Catalog number: 4366597) with TaqMan miRNA specific assays (Catalog number: 4427975). We follow all instructions from the manufacturers, which can be found in the user manuals. The following is a summary that includes precise numerical values for optimized reaction volumes and conditions.

Prepare a 5X stock concentration of all TaqMan miRNA specific reverse transcriptase (RT) primers. Vortex and spin-down all reaction components on a tabletop centrifuge. For each miRNA target: separately prepare a target-specific cDNA and miR-39 specific cDNA reaction using 1 μg of total miRNA for each reaction. Each master mix is created by combining the following components per reaction: 0.15 μL of dNTP Mix, 0.19 μL of RNase Inhibitor, 1.5 μL of 10X RT Buffer, 1 μL of Multiscribe Reverse Transcriptase, and 3 μL of the 5X target-specific RT primer, or the 5X miR-39 specific RT primer.

Following master mix preparation, pipette 5.84 μL of the designated master mix and add 8.16 μL of the 122.55 ng/μL miRNA dilution into each corresponding tube for a total volume of 14 μL.

Next, vortex the selected miR-39 spike-in for 30 s and rest it on ice for 60 s. Repeat this process three times. Then, add 1 μL of the miR-39 spike-in to the respective reactions for a total volume of 15 μL. *Please note: the only difference between miRNA target specific and miR-39 specific reactions is the RT probe*.

For the no template control (NTC) reactions, combine 5.84 μL of the appropriate master mix with 9.16 μL of water in place of cDNA. *Please note: each miRNA-specific master mix requires an NTC to confirm specificity during the data analysis phase*.

Centrifuge the tubes at room temperature and remove any bubbles. *If necessary, pop any remaining bubbles with a clean pipette tip, ensuring all bubbles are eliminated*. Transfer the tubes to a thermocycler set to the following conditions: 4 °C for 5 min; 16 °C for 30 min; 42 °C for 30 min; 85 °C for 5 min; hold at 4 °C forever; lid temperature 105 °C.

After thermocycling is complete, either proceed to the next step immediately or store cDNAs at −20 °C within 24 h. Droplet generation and miRNA specific PCR amplification must be performed within one year of cDNA generation.

### 6. Droplet generation and miRNA specific PCR amplification [Figure 2E]:

In this step, cDNAs will be combined with PCR reaction mixes, partitioned into approximately 20,000 nano-droplets, and then PCR amplified. First, ensure pre-formulated TaqMan miRNA Assays (Tm probes) (Applied biosystems Catalogue # 4427975) are diluted to a 20X concentration in water. TaqMan miRNA Tm probes are supplied in a single tube containing 1 probe and two primers. For this protocol: All miRNA target specific probes are labeled with FAM-MGB while the miR-39 specific probe is labeled with VIC-MGB for multiplexed detection.

Prepare the master mixes for each target by thoroughly mixing the ddPCR Supermix for probes (no dUTP) (Bio-Rad Catalog number: 186-3024) by vortexing for 30 s. To make the master mix, add 12.5 μL of ddPCR Supermix, then 1.25 μL of the VIC-labelled miR39 probe and 1.25 μL of the FAM-labelled target-specific probe, to make a final volume of 15 μL of master mix per sample. Vortex the completed mixtures. *Note: we recommend preparing an additional 10% of the master mix to account for any liquid lost during routine handling*. Vortex each master mix and then centrifuge with a tabletop centrifuge; pipette 15 μL into the appropriate reactions.

Centrifuge the cDNA at 500× g for 30 s. Transfer 5 μL of the appropriate cDNAs into each reaction (5 μL of target-specific cDNA and 5 μL of cDNA of corresponding sample’s miR-39 reaction), resulting in a final volume of 25 μL for each multiplex reaction. Make a negative control (NC) reaction by combining 10 μL of water with 15 μL of the above master mix. Vortex for 30 s, then centrifuge and remove air bubbles with clean pipette tips.

Next, in a sterile trough, pour enough droplet generator oil to generate droplets for all samples. Place the droplet generator plate into the droplet generator case, ensuring that the case is fully closed and that the plate is positioned correctly. Using a multi-channel pipette, gently mix and transfer 20 μL of the sample plus the master mix solution to the droplet generator plate. *Note: take care not to introduce bubbles; always pipette the sample into the plate first, followed by the droplet generator oil. If there are empty wells in a row of the DG8 cartridge, this results in failed droplet generation. If necessary, load 25 μL of the buffer control for probes into any empty well of the DG8 cartridge*.

Using a multi-channel pipette, transfer 70 μL of droplet generator oil (Bio-Rad Catalog number: 1863005) from a sterile trough into the wells of the “oil” row on the droplet generator cassette, taking care not to introduce bubbles. Place the rubber gasket over the top of the droplet generator plate, ensuring that it is fully secure. Run the DG8 droplet generator according to the manufacturer’s instructions.

Following droplet generation, aspirate 40 μL of the droplets by slowly withdrawing the pipette from the bottom of the well at a 35-degree angle over 5 s. *Note: handle the droplets with extreme care to avoid damaging them and do not freeze*. Transfer the droplets into a deep-well 96-well plate (Bio-Rad catalog number: 12001925) by touching the pipette tip to the side of the well, about halfway down. Transfer droplets into the well slowly for 5 s, taking care not to introduce bubbles.

Seal the 96-well plate using the pierceable foil and Bio-Rad PX1 PCR Plate Sealer according to the manufacturer’s instructions. In short, place a foil seal (Bio-Rad Catalog number: 1814040) with the red line facing away from the wells of the plate, ensuring that only one seal is used. *Ensure that the plate sealer block is at room temperature, then* place the 96-well plate on the plate sealer block, and cover it with the plate sealer frame and apply pressure at 180 °C for 5 s. C*heck that the foil is properly sealed by looking for circles etched in the foil where the well openings are located*.

Immediately following the plate sealing process, transfer the deep well 96-well plate into the thermocycler set to the following conditions: 95 °C for 10 min (ramp 2 °C/s); 94 °C for 30 s (ramp 2 °C/s); 60 °C for 1 min (ramp 2 °C/s); repeat at step 2, 39 times; 98 °C for 10 min (ramp 2 °C/s); hold at 4 °C indefinitely; lid temperature: 105 °C. Following PCR, the droplets may be stored at 4 °C for no more than 24 h. Do not freeze and proceed to droplet reading and data analysis.

### 7. Droplet reading and data analysis [Figure 2F]:

After PCR is performed on the droplets, let the 96-well plate rest at room temperature for 5 min prior to transferring to the QX200 Droplet Reader. Follow the instructions for the Bio-Rad QX200 Droplet Reader using the official *Bio-Rad QX200 Droplet Reader Instruction Manual*. The manual provides detailed guidance on the setup, operation, and maintenance of the instrument, including step-by-step procedures, safety considerations, and troubleshooting tips. It is essential to read and follow the instructions carefully to ensure best practices and obtain accurate results. For specific questions or further assistance, contact Bio-Rad directly or consult their technical support resources.

Follow the instructions in the *Bio-Rad QuantaSoft Software Instruction Manual* for data analysis using Bio-Rad QuantaSoft Software. The manual is a comprehensive guide and provides step-by-step instructions for data analysis, including data import, setting analysis parameters, generating reports, and interpreting results. Follow the instructions provided in the manual carefully to ensure accurate and reliable data analysis. If you have specific questions or need further assistance, contact Bio-Rad directly or consult their technical support resources for expert guidance.

Gating in ddPCR is important for accurate and reproducible data analysis, as it allows the discrimination of positive from negative droplets, quantification of target molecules, removal of false positives/negatives, and data normalization. Setting appropriate thresholds ensures reliable and precise results and minimizes background noise and technical artifacts in ddPCR experiments.

Gating ddPCR results requires several steps. First, use the fluorescence channels to detect the target of interest (FAM channel) and the spike-in, reference control marker, miR-39 (VIC channel). Next, establish gating thresholds based on the fluorescence signals to distinguish positive and negative droplets. This can be done manually or with the automated algorithms provided by the analysis software. Apply the gating thresholds to classify droplets into positive and negative populations: positive droplets contain the target of interest and negative droplets do not. Finally, review the gating strategy by inspecting the data to ensure accurate identification of the positive, dual positive, and negative droplet populations.

**Figure 4.**
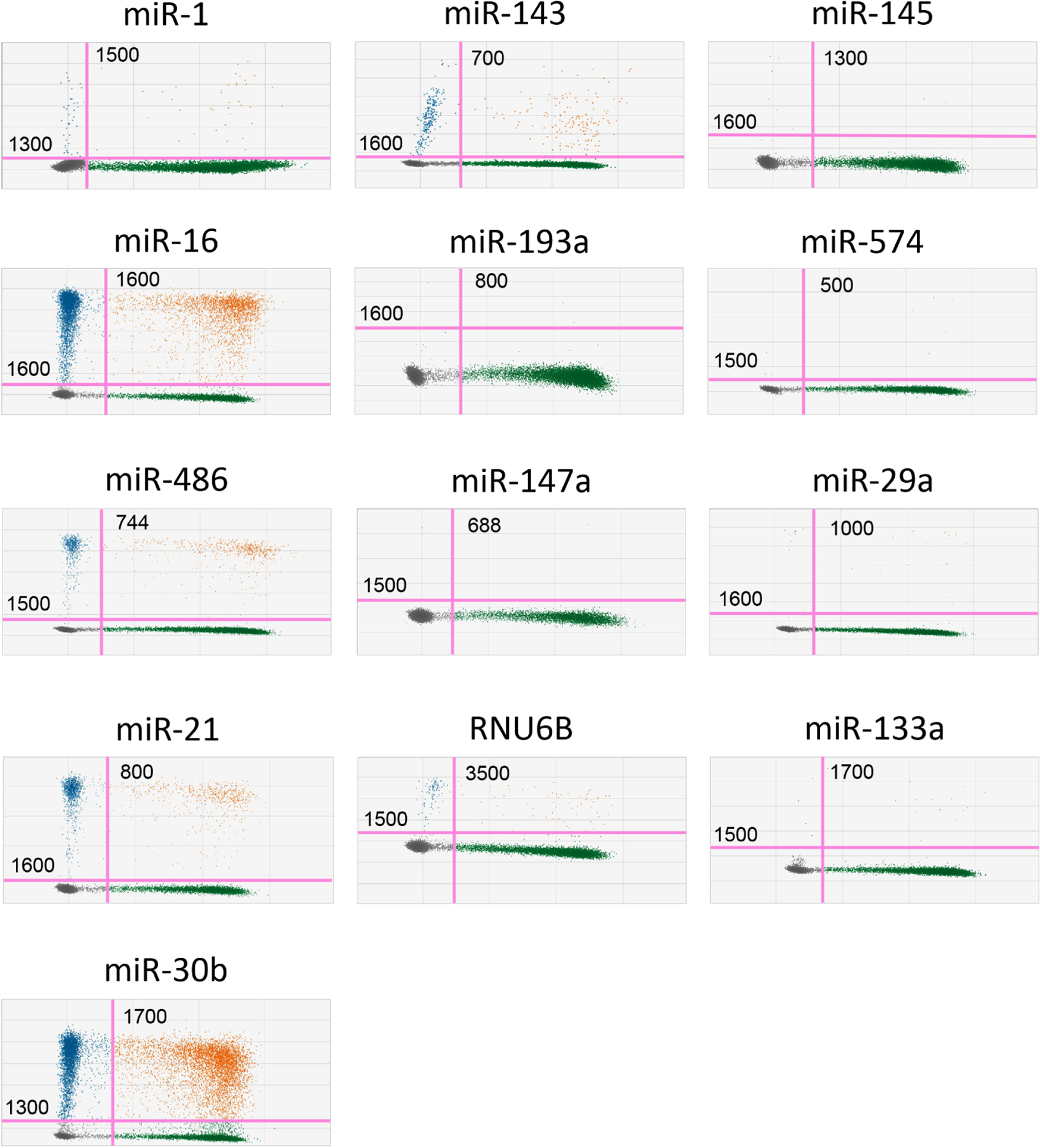
Representative 2-dimensional ddPCR plots of cardiovascular disease related microRNAs. ddPCR plots are separated into 4 quadrants representing FAM(−), VIC(−) events (lower left), FAM(−), VIC(+) events (lower right), FAM(+), VIC(−) events (upper left), and FAM(+) VIC(+) events (upper right). The analysis allows for quantification of total copy numbers, as well as a ratio of the microRNA of interest specific events [FAM(+), VIC(+)] normalized to the spike-in of miR39, represented by FAM(−), VIC(+) events.

Figure 4 contains all representative 2-dimensional plots for each of the following cardiovascular-related miRNA targets. The gating strategy is defined on the X and Y axis of each purple threshold line. These values are held constant within each reaction and are: miR-1 FAM 1300 VIC 1500, miR-133a FAM 1500 VIC 1700, miR-143 FAM 1600 VIC 700, miR-145 FAM 1600 VIC 1300, miR-16 FAM 1600 VIC 1600, miR-193a FAM 1600 VIC 800, miR-21 FAM 1600 VIC 800, miR-29a FAM 1600 VIC 1000, miR-30b FAM 1300 VIC 1700, miR-574 FAM 1500 VIC 500, miR-147a FAM 1500 VIC 688, miR-486 FAM 1500 VIC 744, RNU6B FAM 1500 VIC 3500.

Supplementary Figure 2 contains all representative 2-dimensional plots of each miRNA specific NTC and NC control. Utilizing the gating strategy described above should result in little to no positive droplets being detected in the FAM or VIC fluorescence channels.

For data analysis: Confirm that the number of droplets exceeds 10,000 events and there is little to no fluorescence signal in any of the NTC or NC internal controls. If under 10,000 droplets, repeat from step 6: droplet generation. If a signal appears in the NC and not the NTC, repeat the process from step 5: cDNA generation. If a fluorescence signal is detected in both the NTC and NC, repeat the process from step 3: small RNA quantification and concentration normalization.

After confirming the above and adjusting the thresholds to the described settings, record the copy numbers for each miRNA target and their respective miR-39 values. Apply the Ratio Scale to all miR-39 copy numbers using the below equation, then divide the individual target copy number by its respective ratio scale-corrected miR-39 value. Perform a congruence transformation to normalize miR-39 values by dividing each miR-39 value by the square root of the sum of squares of all the values obtained from each assay (at least three values are required). Below is a representative equation for a congruence transformation of a matrix of values.


$$\frac{ax_{1}\text{,}\;bx_{1}\text{,}\;cx_{1}\text{,}\ldots nx_{1}}{\sqrt{\sum \left(ax_{1}^{2}+bx_{1}^{2}+cx_{1}^{2}+\ldots nx_{1}^{2}\right)}}$$

An example using representative data is: Patient 1 miR-X: 100; miR-39: 1,010 / Patient 2 miR-X: 94; miR-39: 1,203 / Patient 3 miR-X: 114; miR-39: 973. The first step is to square all miR-39 values (1,010^2^, 1,203^2^, 973^2^) = (1,020,100, 1,447,209, 946,729). Next, perform the sum of the squares (1,020,100 + 1,447,209 + 946,729) = (3,414,038). Then calculate the square root of the sum of squares: √ (3,414,038) = (1,847.71). Divide each value by the square root of the sum of squares (1,010/1,847.71, 1,203/1,847.71, 973/1,847.71) = (0.546, 0.651, 0.526). Finally, divide each microRNA target value (miR-X) by its respective ratio scale corrected miR-39 value (100/0.546, 94/0.651, 114/0.526) = (183.15, 144.39, 216.73). These are the miRNA values to report for each patient.

Ratio Scale normalization is a method used to correct target values to a common scale^[10]^. This is because the ratios among the intervals between numbers are not affected by congruence transformations, which makes it useful for comparing values from different datasets using different plates or miR-39 spike-in lots.

Table 1 details the expected values of all miRNA targets in healthy individuals with no significant medical history. While no statistical differences were detected between healthy males and females, Supplementary Table 1 has all levels demarcated by sex. These tables are provided to assist in the independent corroboration of results using this method.

## DISCUSSION

Accurate quantification of nucleic acids, particularly miRNAs, from blood plays a pivotal role in understanding disease mechanisms, identifying biomarkers, and developing diagnostic approaches. However, ensuring the reliability and reproducibility of quantitative results necessitates standardized protocols for blood collection, plasma processing, cryo-storage, miRNA isolation, reverse transcription, droplet generation, PCR amplification, fluorescence reading, and data analysis. By standardizing these steps and implementing appropriate quality control measures, researchers and clinicians can enhance the robustness and validity of circulating miRNA quantification. To assist independent corroboration of conclusions, this protocol provides comprehensive disclosure of all relevant experimental details required to rapidly advance the fields of molecular diagnostics and personalized medicine.

Each year in the United States, nearly 10,000 people die from aortic aneurysms, and an additional 16,000 die from complications associated with aortic disease. Aortic aneurysm is the 17th leading cause of death for those over age 65^[11,12]^. Discovery most often occurs during evaluation of unrelated problems. This diagnostic process is inherently sub-optimal, leaving many undiagnosed and at risk for catastrophic complications such as aortic rupture or dissection. During pathological progression, we have demonstrated that miRNAs are secreted from pathological tissues into the circulation^[13]^, and levels correlate linearly to aortic diameter measurements^[3,14]^. Moreover, different etiological subtypes generate unique miRNA signatures^[3]^. Taken together, this suggests that measuring circulating miRNAs may be used to diagnose, locate, and track aortic aneurysm progression in the general population, ultimately informing physicians of optimal timing for surgical intervention.

Standardizing the process of blood draw and use of anticoagulants is crucial for downstream PCR analysis. Different anticoagulants can impact the quality and quantity of nucleic acids isolated from blood, affecting the accuracy and reliability of PCR results^[15]^. In this protocol, we demonstrate that EDTA is compatible with nucleic acid isolation methods and ddPCR assays. Additionally, EDTA is an effective anticoagulant that preserves nucleic acids, including miRNAs, by chelating divalent cations necessary for nuclease activity^[16]^. This helps prevent the degradation of nucleic acids during plasma processing. Heparin, however, should be avoided as it can inhibit PCR amplification^[17]^. This underlines the importance of standardizing blood collection methods. Standardizing blood draw and anticoagulant use ensures a regulated foundation on which to proceed and achieve reproducible miRNA quantification.

The separation of blood into cellular and liquid layers in the presence of an anticoagulant through centrifugation allows for the isolation of plasma. Blood plasma provides a rich source of miRNAs. It offers a non-invasive and easily accessible sample type, collected through standard venipuncture techniques, which makes it convenient for use in diagnostic testing. Moreover, plasma is stable, abundant, and compatible with this protocol. Additionally, the systemic representation of plasma allows for longitudinal monitoring, providing broader insights into overall health status. While miRNAs may be recovered from serum, the number of recovered miRNAs will differ significantly. For example, a recent study comparing miRNA recovered from plasma vs serum of acute myocardial infarction patients revealed differential expression patterns^[18]^. Therefore, we recommend the use of plasma for this protocol.

During the plasma isolation process, it is important to monitor for hemolysis as it likely indicates sample degradation or mishandling^[19]^. Use of a large bore (e.g., 18 gauge) collection needle, and gentle handling during processing can limit hemolysis. Hemolysis can compromise the accuracy, specificity, and reliability of results due to the presence of PCR inhibitors, contamination with genomic DNA, and distortion of target nucleic acid concentration^[19]^. Hemolytic samples should be excluded from analysis.

Proper handling and transfer of plasma to sterile, nuclease-free tubes ensure preservation for quantitative ddPCR analysis. Snap freezing rapidly preserves samples, typically using liquid nitrogen or a slurry or dry ice and isopropanol^[20]^. This method must be performed when preserving plasma and prior to downstream storage of isolated nucleic acids, as it helps to prevent the formation of large ice crystals, which can damage the nucleic acids. Cryo-preservation of plasma at temperatures of −80 °C or below helps inhibit enzymatic activity and chemical reactions that lead to nucleic acid degradation. To maintain miRNA integrity during storage and handling, it is important to limit freeze-thaw cycles. Each cycle can cause significant nucleic acid degradation or fragmentation. Storing samples in aliquots eliminates the negative effects of freeze-thaw cycles.

During the freeze-thaw process, nucleic acids are vulnerable to damage from changes in pH, physical shearing, and denaturation^[21]^. Slowly thawing samples minimizes damage by allowing for gradual pH change, reducing the rate of ice crystal formation, and allowing the nucleic acids to refold, reducing denaturation. Using sterile, nuclease/pyrogen-free water/buffers/tubes, freezing the sample rapidly, thawing them slowly, and avoiding repeated freeze-thaw cycles are all essential components of reproducibility.

There are myriad ways to isolate miRNAs from plasma, each with its own capacities, advantages, disadvantages, and idiosyncrasies. For example, we have found that the phenol-chloroform extraction method is incredibly cost-effective and efficient for the isolation of messenger (mRNAs)^[13,22]^. However, this method does not efficiently isolate small RNAs from plasma. Adding different components, such as glycogen, can mitigate this to a degree, but because circulating miRNA levels are relatively low, these methodologies cannot yield enough for direct quantification by this method. While pre-amplification has been used in the past, these additional modifications result in inevitable variations, making results less comparable across studies.

For this protocol, we have chosen the miRNeasy Serum/Plasma Advanced Kit from Qiagen, which has previously been reported to be among the optimal kits to isolate miRNA from plasma^[9]^. The kit utilizes a silica-based membrane and chaotropic salt to selectively bind and elute small RNAs. Although it is not the most cost-efficient method available, it is comprehensively effective, standardized, and can be automated.

The importance of beginning analysis with even amounts of isolated miRNA cannot be overstated. Isolation efficiency depends largely upon handling and the presence of contaminants. There are several different ways to measure and normalize the amount of total miRNA. These include volume corrections following analysis by spectroscopy, electrophoresis, and/or nucleic acid dyes (as in the Qubit Assay).

The Invitrogen Qubit miRNA assay utilizes non-sequence specific nucleic acid dyes that emit fluorescence only when bound to miRNA (17–25 bp), offering an accurate and selective method for quantification. The fluorescence signal is proportional to the amount of miRNA. Samples are compared to a standard curve, making it a direct quantification rather than a surrogate measure as with spectroscopy. The dye is highly selective for intact miRNA over ribosomal (rRNA) or larger mRNAs (> 1,000 bp). The kit provides accurate results for miRNA concentrations ranging from 0.025 to 150 ng/μL, with a detection range of 0.5–150 ng in volumes between 1 μL and 20 μL. Furthermore, fluorescence measurements are more sensitive, allowing for reduced noise levels. In comparison to traditional UV absorbance methods, this approach offers improved accuracy by mitigating overestimations caused by contaminants such as salts, solvents, detergents, proteins, and free nucleotides^[23]^.

Multiple studies have compared different quantification methods of total miRNA^[24,25]^. Garcia-Elias and colleagues directly compared the Infinite^®^ 200 PRO Nanoquant and Nanodrop 2000 spectrophotometers, and the Agilent 2100 Bioanalyzer PicoChip and SmallChip, and the Qubit^®^ 2.0 Fluorometer. Their results showed that the spectrophotometric measurements overestimated RNA concentration due to the detection of contaminants. These results are consistent with those of Wright *et al*., who also found that the Nanodrop consistently reported higher concentrations of RNA compared to the BioAnalyzer and the Qubit. Thus, we strongly recommend the use of the Qubit, where available.

RNA Integrity Number (RIN) is a score that measures the intactness of isolated RNA molecules -intactness, in this context, is commonly called integrity or quality. RIN was developed as a quality assessment metric based on nucleic acid length^[25]^. The RIN value is based on the electrophoretic profile of RNA from cells or tissue, which contain classes of ribosomal RNA such as 18 s and 28 s rRNA. These are used for the RIN algorithm and can be problematic in this application for several reasons. First, plasma and serum have inherently lower populations of 18s and 28s rRNA and so typically provide lower RIN values in this context^[25]^. Second, isolated miRNA are enriched for small RNA (< 25 bp), so the RIN value is irrelevant. For these reasons, RIN cannot be reliably used to determine small RNA quality. The Qubit miRNA assay specifically targets a narrow and specific nucleic acid size-length range. In this protocol, if measured values fell outside the range reported in Figure 3, results likely indicated degradation or contamination. We suggest that compromised samples with concentrations outside these ranges must be excluded from subsequent analysis.

While standardization of starting input is indispensable, inherent constraints and natural variability necessitate additional corrections to account for differences in reaction efficiencies and overall quantification. Traditionally, internal referent normalization accounts for these variations. It involves comparing target values to a reference gene. The ideal reference gene should be unaffected by the experimental treatment or pathology and should be expressed at a similar level to that of the target to preserve the linear dynamic range of detection. Several “housekeeping” mRNAs are generally accepted and widely used (e.g., GAPDH, beta-actin)^[26]^. The former cannot function in this protocol, as quantification would require different chemistries.

Several small RNAs, like miR-16 and RNU6B, have been identified to be stable in specific pathologies^[27]^. Unlike miRNAs, small-nucleolar RNAs, like RNU6B, are subject to entirely different regulatory processes, making normalization less relevant. Nevertheless, none of the proposed small RNA housekeeping molecules have been demonstrated to be stably expressed across cardiovascular diseases/co-morbidities. This problem is multiplied when one considers complex pathologies with many subtypes and genetic preconditions, such as aortic aneurysm disease, making it easy to see that previous methods are not feasible in this context.

To avoid the issues described above, we developed a method for normalization to a miRNA spike-in using the synthetic, exogenous nucleic acid molecule miR-39. It is prepared and validated prior to being added to samples to serve as a reference for data normalization and control for inevitable variations in all experiments. These controls help monitor experimental efficiency and accuracy and allow for the normalization of gene expression levels between specimens.

The specificity of target amplification is enhanced when loop-specific cDNA generation is used, as in TaqMan miRNA assays, to convert miRNAs into complementary DNA (cDNA) for subsequent PCR amplification. The chemistry involves a small-RNA specific, stem-looped oligonucleotide primer that specifically hybridizes to the 3’ end of each target miRNA. The resulting cDNA is then amplified and quantified using TaqMan PCR primers and probes.

Hydrolysis probe chemistry is another essential component of miRNA quantification. Hydrolysis probes bind to the target miRNA sequence and have a fluorescent reporter dye at one end and a quencher molecule at the other. During PCR amplification, the probe binds specifically to the target miRNA sequence, and when the RNA polymerase reaches the probe during the amplification process, it separates the reporter dye from the quencher. This reaction is observed as a fluorescent signal, which is detected and quantified in each droplet of the ddPCR system.

When performing this protocol, it is important to adhere to the most recent ddPCR MIQE guidelines^[28]^. In accordance with these guidelines, all TaqMan small RNA assays have been tested and optimized for specificity, reproducibility, linear dynamic range, sensitivity, and efficiency, which are all available via ThermoFisher. This enhances overall reproducibility and consistency when comparing analyses across laboratories.

Droplet generation is a critical step in this workflow. Microfluidics technology partitions the PCR reaction into thousands of droplets, each acting as a separate reaction chamber. This enables the amplification and quantification of miRNAs with high sensitivity and accuracy.

Plasma miRNAs hold great promise as potential diagnostic markers due to their stability, ease of collection, and association with various diseases. However, there are certain limitations that need to be considered. First, the presence of abundant non-specific miRNAs and other extracellular RNA species in plasma can hinder the specificity and sensitivity of miRNA detection. This necessitates the use of stringent purification methods and the inclusion of appropriate controls to minimize false-positive results. Additionally, the variability in miRNA expression patterns among individuals and the lack of standardized reference genes make it challenging to establish universally applicable diagnostic thresholds. The use of an exogenous spike-in control mitigates this challenge. Furthermore, while ddPCR offers enhanced sensitivity and precision, it is limited by the number of miRNA targets that can be simultaneously analyzed in a single reaction due to the partitioning of the sample into droplets. Overcoming these challenges requires further optimization of purification techniques, standardization of reference genes, and the development of multiplexing strategies to fully exploit the diagnostic potential of plasma miRNAs and ddPCR in clinical settings.

The accurate quantification of miRNAs from blood samples is a crucial aspect of molecular diagnostics and research. By following standardized protocols for blood collection, preservation, and processing, researchers can ensure the integrity and stability of nucleic acids, which is essential for reliable PCR analysis. Standardizing the use of anticoagulants, minimizing freeze-thaw cycles, and incorporating spike-in controls are critical steps in achieving accurate and reproducible miRNA quantification. Furthermore, the utilization of advanced techniques such as loop-specific cDNA generation and droplet partitioning through digital PCR provides enhanced sensitivity, precision, and multiplexing capabilities for miRNA analyses. By implementing these methodologies and adhering to rigorous quality control measures, researchers can overcome technical limitations, enhance the accuracy of miRNA quantification, and pave the way for improved diagnostics.

## Supplementary Material

Supplementary Material

## Figures and Tables

**Figure 2.**
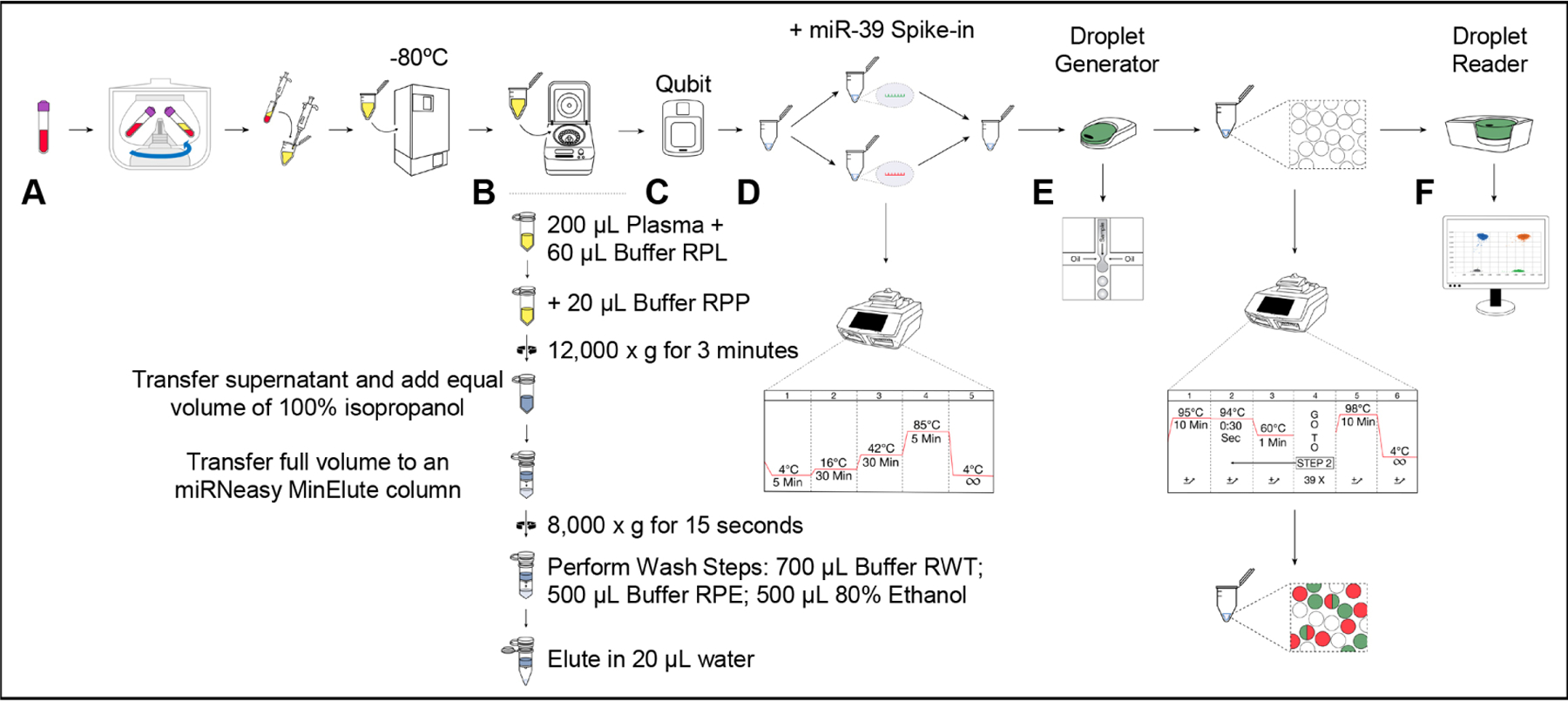
Schematic diagram of the microRNA quantification workflow. (A) Blood collection, plasma isolation, and long-term storage. Peripheral venous blood is collected in BD Vacutainer^®^ EDTA-coated tube and plasma is separated by centrifugation (2,500× g, 15 min, room temperature). Plasma supernatant is removed and centrifuged again and supernatant is aliquoted for storage at −80 °C. (B) Small RNA Isolation from Plasma. microRNA is isolated according to the manufacturer’s instructions of the Qiagen miRNeasy Serum/Plasma Advanced Kit. (C) Small RNA quantification and concentration normalization. The concentration (ng/μL) of eluted microRNA is quantified using the Qubit microRNA assay, according to manufacturer’s instructions. Concentration is subsequently adjusted to 122.55 ng/μL for all samples. (D) Spike-in addition and microRNA specific cDNA generation. miR-39 is exogenously spiked into the microRNA sample, and target-specific cDNA generation is performed utilizing TaqMan microRNA Reverse Transcription Kit following manufacturer’s instructions. miR-39-specific and target-specific cDNA generation are performed separately to avoid cross-reactivity. (E) Droplet generation and microRNA specific PCR amplification. miR-39 cDNA and target-specific cDNA are mixed together with ddPCR supermix (no DUTP), and respective FAM and VIC miR-39 and target-specific cDNA amplification primers. Samples are transferred to Bio-Rad droplet generator microfluidic chips alongside droplet generator oil and transferred to the QX200 Droplet Generator. Droplets are transferred to a deep-well PCR plate, followed by PCR amplification. (F) Droplet reading and data analysis. The samples are read by the Bio-Rad QX200 Droplet Reader and analyzed with Bio-Rad QuantaSoft software. All appropriate instructions in user manuals are adhered to.

**Table 1.** Referent plasma microrna levels in healthy people

Abbreviation	Assay ID	Accession number	Sample size *n* (female, male)	Age (Avg. ± SEM)	Mean normalized concentration (Avg. ± SEM)
miR-1	002222	MIMAT0000416	*n* = 26 (13, 13)	37.96 ± 3.18	5.2817 ± 1.4331
miR-133a	002246	MIMAT0000427	*n* = 20 (11, 8)	40.05 ± 3.54	7.7218 ± 3.2159
miR-143	002249	MIMAT0000435	*n* = 26 (13, 13)	39.38 ± 3.37	56.1333 ± 11.0116
miR-145	002149	MIMAT0004601	*n* = 26 (13, 13)	37.96 ± 3.18	2.9591 ± 1.0029
miR-16	000391	MIMAT0000069	*n* = 26 (13, 13)	39.46 ± 3.19	1,786.6109 ± 547.6928
miR-193a	002250	MIMAT0000459	*n* = 26 (13, 13)	37.96 ± 3.18	3.5471 ± 1.2057
miR-21	000397	MIMAT0000076	*n* = 26 (13, 13)	39.03 ± 3.26	332.7083 ± 83.2669
miR-29a	002112	MIMAT0000086	*n* = 26 (13, 13)	39.53 ± 3.34	37.3906 ± 12.4067
miR-30b	000602	MIMAT0000420	*n* = 26 (12, 14)	41.00 ± 2.67	4,895.2872 ± 1,342.2487
miR-574	002349	MIMAT003239	*n* = 26 (12, 14)	40.57 ± 3.24	7.1525 ± 1.5354
miR-147a	000469	MIMAT0000251	*n* = 25 (12, 13)	41.56 ± 3.21	0.2082 ± 0.0646
miR-486	001278	MIMAT0002177	*n* = 26 12, 14)	40.57 ± 3.24	785.2370 ± 292.3945
RNU6B	001973	NR_004394	*n* = 26 (11, 15)	39.43 ± 3.09	53.0385 ± 26.6712

## Data Availability

All relevant data are within the manuscript and its Supplementary Materials.
