# Supplementary material for: Plasma microrna quantification protocol
Source: Vessel Plus. Author manuscript; Available in PMC 2024 Mar 5. (PMC10914336; doi:10.20517/2574-1209.2023.69)
Supplement: Supplementary Material [file NIHMS1959438-supplement-Supplementary_Material.pdf]

Supplemental Figure 1

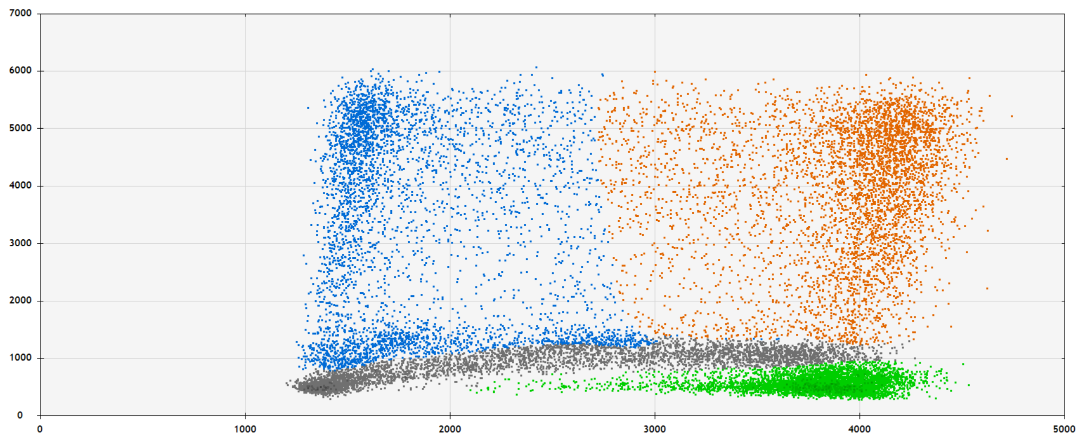

Supplemental Figure 1. Representative 2-dimensional ddPCR plot of cDNA generated with miR-39 specific probe and target-specific probe in a single tube. Failure to separate the cDNA as shown in Figure 2D into two tubes where miR-39 specific-cDNA generation occurs separate to target-specific cDNA generation results in greater noise at the final steps of ddPCR droplet reading.

Supplemental Figure 2

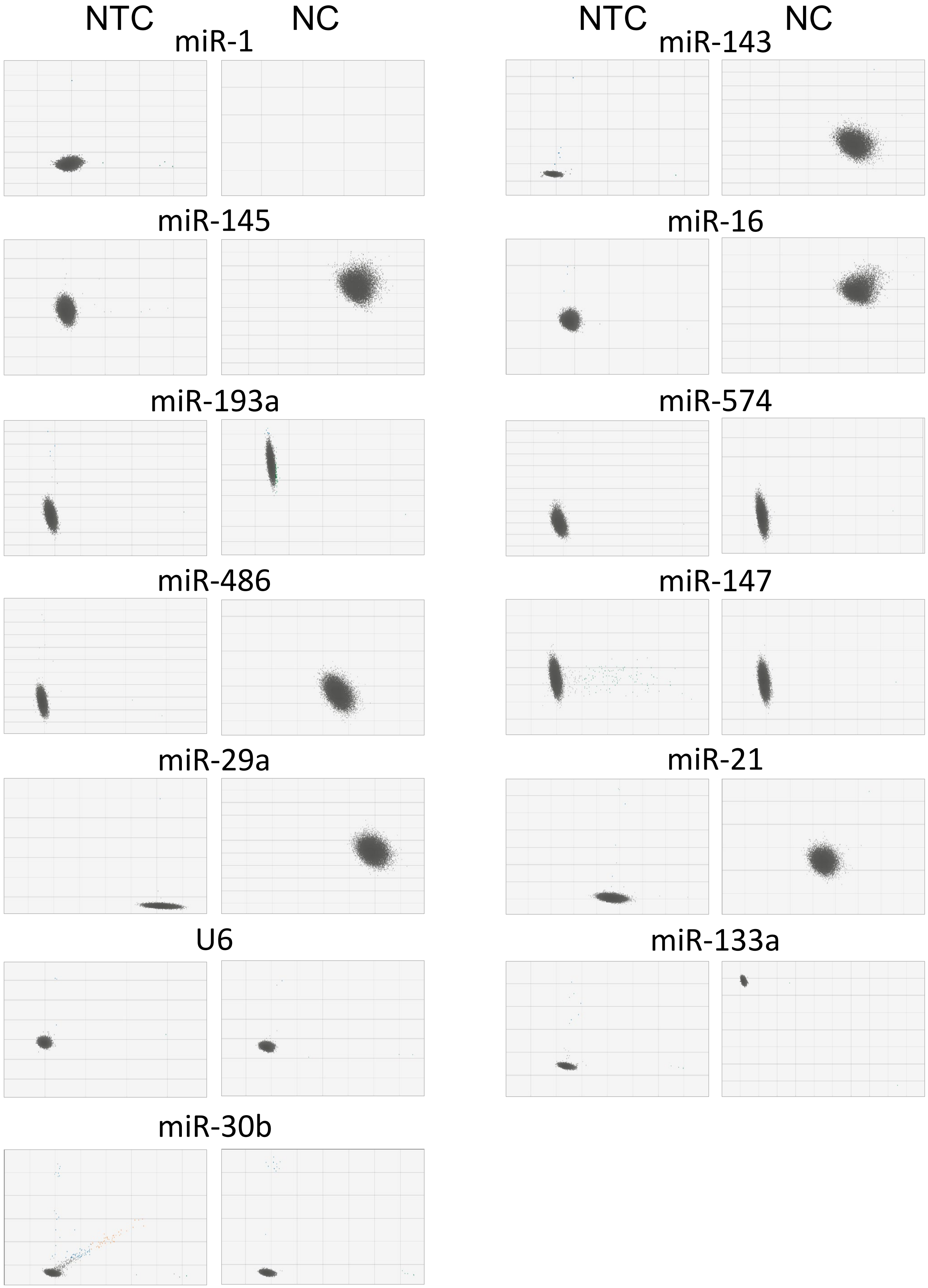

Supplemental Table 1. Plasma MicroRNA levels in Healthy Males and Females.

Abbreviation	Assay ID	Accession Number	Sample Size n (Female, Male)	Age (Avg. ± SEM)	Mean Normalized Concentration (Avg. ± SEM)
Total miR-1	002222	MIMAT0000416	n = 26 (13, 13)	37.96 ± 3.18	5.2817 ± 1.4331
Female miR-1	-	-	n=13	37.00 ± 4.80	4.7064 ± 1.5691
Male miR-1	-	-	n=13	39.61 ± 3.91	5.8570 ± 2.4547
Total miR-133a	002246	MIMAT0000427	n = 20 (11, 8)	40.05 ± 3.54	7.7218 ± 3.2159
Female miR-133a	-	-	n=11	38.68 ± 4.95	8.1352 ±4.5497
Male miR-133a	-	-	n=8	42.37 ± 5.77	4.2197 ±1.5076
Total miR-143	002249	MIMAT0000435	n = 26 (13, 13)	39.38 ± 3.37	56.1333 ± 11.0116
Female miR-143	-	-	n=13	39.64 ± 5.29	65.2608 ± 19.3089
Male miR-143	-	-	n=13	39.61 ± 3.91	47.0057 ± 10.8863
Total miR-145	002149	MIMAT0004601	n = 26 (13, 13)	37.96 ± 3.18	2.9591 ± 1.0029
Female miR-145	-	-	n=13	37.00 ± 4.80	2.2596 ± 0.4452
Male miR-145	-	-	n=13	39.61 ± 3.91	3.6586 ±1.9777
Total miR-16	000391	MIMAT0000069	n = 26 (13, 13)	39.46 ± 3.19	1786.6109 ± 547.6928
Female miR-16	-	-	n=13	37.00 ± 4.80	1340.2310 ± 343.4842
Male miR-16	-	-	n=13	39.61 ± 3.91	2391.6471 ± 1082.1704
Total miR-193a	002250	MIMAT0000459	n = 26 (13, 13)	37.96 ± 3.18	3.5471 ± 1.2057
Female miR-193a	-	-	n=13	37.00 ± 4.80	2.7907 ± 1.2489
Male miR-193a	-	-	n=13	38.50 ± 4.18	4.3034 ± 2.0982
Total miR-21	000397	MIMAT0000076	n = 26 (13, 13)	39.03 ± 3.26	332.7083 ± 83.2669
Female miR-21	-	-	n=13	41.95 ± 4.36	287.2664 ± 102.0324
Male miR-21	-	-	n=13	38.7 ± 4.70	378.1503 ± 134.6638
Total miR-29a	002112	MIMAT0000086	n = 26 (13, 13)	39.53 ± 3.34	37.3906 ± 12.4067
Female miR-29a	-	-	n=13	41.76 ± 4.65	43.4734 ± 21.0880
Male miR-29a	-	-	n=13	38.7 ± 4.70	31.0167 ± 10.7500
Total miR-30b	000602	MIMAT0000420	n = 26 (12, 14)	41.00 ± 2.67	4895.2872 ± 1342.2487

Female miR-30b	-	-	n=12	38.29 ± 4.25	4235.9521 ± 1039.5741
Male miR-30b	-	-	n=14	43.71 ± 3.34	5067.9667 ± 2311.7693
Total miR-574	002349	MIMAT003239	n = 26 (12, 14)	40.57 ± 3.24	7.1525 ± 1.5354
Female miR-574	-	-	n=12	44.55 ± 4.47	7.5363 ± 2.5904
Male miR-574	-	-	n=14	36.50 ± 4.49	7.8841 ± 2.3675
Total miR-147a	000469	MIMAT0000251	n = 25 (12, 13)	41.56 ± 3.21	0.2082 ± 0.0646
Female miR-147a	-	-	n=12	44.55 ± 4.47	0.1902 ± 0.1002
Male miR-147a	-	-	n=13	38.07 ± 4.54	0.1955 ± 0.0760
Total miR-486	001278	MIMAT0002177	n = 26 (12, 14)	40.57 ± 3.24	785.2370 ± 292.3945
Female miR-486	-	-	n=12	44.55 ± 4.47	532.5451 ± 178.0567
Male miR-486	-	-	n=14	36.50 ± 4.49	1077.4585 ± 525.9436
Total RNU6B	001973	NR_004394	n = 26 (11, 15)	39.43 ± 3.09	53.0385 ± 26.6712
Female RNU6B	-	-	n=11	43.70 ± 4.68	15.2799 ± 3.8965
Male RNU6B	-	-	n=15	37.37 ± 4.11	81.6221 ± 45.3507

Supplemental File 1

miR-39 Spike-In Validation

miR-39 Large Volume Dilution Preparation

Materials

- cel-miR-39-3P Mimic (mc10956, Life Technologies)
- Qubit microRNA Assay Kit (Ref #: Q32881, Thermo Fisher Scientific)
- RNAse Free, DPEC Free Water (Cat #: 02-0201-0500, VWR Life Science)

Procedure

1. Resuspend 5 nmol of cel-miR-39-3P mimic in 200 microliters of water to create a 25 nM solution.
2. Prepare a 0.5 μM dilution (1:50) by pipetting 1-part 25 nM cel-miR-39-3P mimic and 49 parts water.
3. Calculate concentration (in ng/μL) using the Qubit microRNA Assay kit
4. Create a 1:10 dilution series starting from a 1,000 ng/μL cel-miR-39-3p mimic dilution until reaching a concentration of 0.0001 ng in water.
5. Label the tubes with the miR-39 concentration, assign a lot number, and date.
6. Store at -80°C until use.

miR-39 Spike-In Validation

cDNA Generation

Materials

- TaqMan MicroRNA Reverse Transcription Kit (Thermo Fisher Scientific) (Cat #: 4366597)
- RNAse Free, DPEC Free Water (Cat #: 02-0201-0500, VWR Life Science)
- cel-miR-39-3p, 4427975, Assay ID: 000200, Accession #: MIMAT0000010.

Procedure

1. Prepare a Master Mix with the following components.

Component	Single Reaction Volume
Dntp Mix	0.15 µL
RNase Inhibitor	0.19 µL
10X RT Buffer	1.5 µL
Multiscribe Reverse Transcriptase	1 µL
cel-miR-39-3p VIC_MGB labeled (20X)	0.75 µL
RNAse Free, DPEC Free Water	10.41 µL
**Total**	**14 µL**

2. Pipette **14 µL** of each **master mix** into the appropriate tubes.
3. Pipette **1 µL** of the cel-miR-39-3p spike-in dilution into the appropriate tubes. *Before adding the spike-in vortex the miR-39 aliquot for 30 seconds and rest in ice for 30 seconds – repeat three times.*
4. Create a No Template Control (NTC) by adding **1 µL** of water to the 14µL of corresponding master mix.
5. Centrifuge each tube with the tabletop centrifuge to collect liquid at the bottom and remove any bubbles. If necessary, pop bubbles with a clean pipette tip. Inspect tubes to ensure all bubbles are removed.
6. Place tubes in the thermocycler.
7. Thermocycler conditions:

   Lid Temperature at 105°C,
   5 minutes at 4°C,
   30 minutes at 16°C,
   30 minutes at 42°C,
   5 minutes at 85°C,
   Hold at 4°C

8. Upon completion cDNA can be stored at -20°C until use.

miR-39 Spike-In Validation

ddPCR

Materials

- ddPCR Supermix for Probes (no dutp)
  (Cat #: 186-3024, BIORAD)
- Droplet Generator Oil for Probes
  (Cat #: 1863005)
- DG8 Cartridges for QX200/QX100
  (Cat #: 1864008)
- Drop Generator Holder (Cat #: 1863051)
- Rubber Gasket (Cat #: 1863009)
- PCR Deep Well Plate (Cat #: 12001925)
- Foil Seal (Cat #: 1814040)
- cel-miR-39-3p, Cat # 4427975, Assay ID 000200, Accession # MIMAT0000010 VIC_MGB labeled.
- RNAse Free, DPEC Free Water (Cat #: 02-0201-0500, VWR Life Science)

Procedure

1. If necessary, prepare 20X Tm probes.
2. Prepare a master mix using the following components:

**Component**	**Single Reaction Volume**
SMX (no dutp)	12.5 µL
cel-miR-39-3p VIC_MGB labeled (20X)	1.25 µL
RNAse Free, DPEC Free Water	6.25 µL
**Total**	**20 µL**

3. Vortex the miR-39 master mix and centrifuge with tabletop centrifuge.
4. Transfer 20 µL of the master mix into each appropriate tube
5. Transfer 5 µL of the appropriate cDNA into each respective tube. The final volume for the reactions will be 25 µL.
6. Create a Blank reaction by combining 20 µL of miR-39 master mix with 5 µL of water.
7. Centrifuge the tubes to remove air bubbles and settle liquid at the bottom of the tube. Check all air bubbles have been removed. Pop any remaining air bubbles with pipette tips.
8. Generate droplets using the Bio-Rad DG8 Droplet Generator following the manufacturer's instructions.

<u>miR-39 Spike-In Validation</u>

9. Using a multichannel pipette, transfer **40 µL** of the droplets into the 96 well, deep-well plate.
10. Seal the 96-well plate using a foil seal and the Bio-Rad PX1 PCR Plate Sealer following the manufacturer's instructions.
11. Place the deep well 96 well plate into a thermocycler.
12. Thermocycler conditions:

    Lid Temperature at 105°C,
    95°C for 10 minutes; ramp 2°C/sec,
    94°C for 30 seconds; ramp 2°C/sec,
    60°C for 1 minute; ramp 2°C/sec,
    Go to 2°C 39 times,
    98°C for 10 minutes; ramp 2°C/sec,
    4°C forever
13. Incubate the plate at room temperature for five minutes.
14. Perform droplet reading using the Bio-Rad QX-200 following the manufacturer's instructions.
15. Proceed to Droplet reading and data analysis steps detailed in the associated technical note.

Supplemental File 2

Plasma microRNA Quantification Protocol

Small RNA Isolation from Plasma and Small RNA Quantification and Concentration Normalization

Materials

- Human plasma
- Qubit microRNA Assay (Ref #: Q32881)
- RNAse Free Water
- miRNEasy serum Plasma Advanced kit (Ref #: 217204)

Procedure

1. Retrieve plasma samples from -80°C and thaw on ice.
2. Use pipette to mix vigorously and transfer 240 µL of plasma into a 2 mL microcentrifuge tube.
3. Centrifuge plasma at 10,000 x g for 10 minutes at 4°C.
4. Transfer 200 µL of plasma supernatant to a new 2 mL microcentrifuge tube.
5. Add 60 µL of Buffer RPL and vortex for 5 seconds, incubate at room temperature for 3 minutes.
6. Add 20 µL of Buffer RPP and vortex for 20 seconds, incubate at room temperature for 3 minutes.
7. Centrifuge at 12,000 x g for 3 minutes at room temperature.
   a. Supernatant should be clear and colorless.
8. Transfer supernatant to a new microcentrifuge tube
   a. Add 1 volume of isopropanol, vortex for 30 seconds.
   b. The volume should be approximately 230 µL.
9. Transfer the entire sample to a RNeasy UCP MinElute column. Centrifuge for 15 seconds at 8,000 x g, discarding the flow through.
10. Add 700 µL Buffer RWT to the RNeasy UCP MinElute column. Centrifuge for 15 seconds at 8,000 x g, discarding the flow through.
11. Add 500 µL of Buffer RPE to the RNeasy UCP MinElute column. Centrifuge for 15 seconds at 8,000 x g, discarding the flow through.
12. Add 500 µL of 80% Ethanol to the RNeasy UCP MinElute column. Centrifuge for 2 minutes at 8,000 x g, discarding the flow through.
13. Place the RNeasy UCP MinElute spin column inside a new 2 mL collection tube. Open lid of the spin column and centrifuge at maximum speed for 5 minutes to dry the membrane, discarding the flow through and collection tube.
14. Place the RNeasy UCP MinElute spin column inside a new 1.5 mL collection tube.
    a. Add 20 µL of RNase-free water directly into the center of the spin column membrane and incubate at room temperature for 10 minutes.
    b. Then, centrifuge for 1 minute at maximum speed to elute the purified small RNA.
15. Perform small RNA quantification using the Qubit microRNA assay.
16. For each sample, dilute small RNA to a concentration of 122.55 ng/µL using the below equations.
    a. The first portion will solve for the full volume of small RNA in water needed to reach a concentration of 122.55 ng/µL.

$$Final\ RNA\ Volume\ (\mu L) = \frac{Qubit\ Concentration\ (ng/\mu L) * \ Original\ RNA\ Volume\ (\mu L)}{122.55\ (\mathrm{ng/\mu L})}$$

    b. Then, the second part will solve for the volume of water needed to add to the original volume of RNA in water in order to convert the total amount of RNA to 122.55 ng/µL.

$$Volume\ of\ Water\ to\ Add\ (\mathrm{\mu L}) = Final\ RNA\ Volume\ (\mathrm{\mu L}) - Original\ RNA\ Vol.\ (\mathrm{\mu L})$$

Plasma microRNA Quantification Protocol

Spike-in addition and miRNA specific cDNA generation

Materials

- Small RNA from previous step
- TaqMan MicroRNA Reverse Transcription Kit (Thermo Fisher Scientific: 4366597)
- miR-39 Spike-in (prepared in previous protocol)
- Target-specific TaqMan RT Primers (5X) (see Table 1 for catalogue numbers)
- miR-39-specific TaqMan RT Primers (5X) (cel-miR-39-3p, 4427975, Assay ID 000200, Accession # MIMAT0000010)

Procedure

1. Prepare 5X probes for any target-specific primers at a higher concentration.
2. Prepare a miR-39-specific and target-specific master mix for each microRNA being measured using the following table as a guide.

Component	***Volume per Reaction***
dNTP Mix	0.15 µL
RNase Inhibitor	0.19 µL
10x RT Buffer	1.5 µL
Reverse Transcriptase Enzyme	1 µL
microRNA-specific RT Primer (5X)	3 µL
Total	**5.84 µL**

3. Pipette **5.84 µL** of the appropriate master mix into the corresponding tubes and add **8.16 µL** of **122.55 ng/ µL** small RNA. For the NTC reaction, add **9.16 µL** of RNase free water in place of the small RNA and water.
4. Before adding the spike-in vortex the miR-39 spike-in for 30 seconds and rest on ice for 60 seconds – repeat three times.
5. Pipette **1 µL** of the selected miR-39 spike-in into the appropriate tubes and record the lot number and corresponding measured miR-39 copy number.
6. Centrifuge each tube with the tabletop centrifuge to collect liquid at the bottom and remove any bubbles. If necessary, pop bubbles with a clean pipette tip. Inspect tubes to ensure all bubbles are removed.
7. Place tubes in the thermocycler.
8. *Thermocycler Conditions*: Lid Temperature at 105°C, 5 minutes at 4°C, 30 minutes at 16°C, 30 minutes at 42°C, 5 minutes at 85°C, Hold at 4°C.
9. Upon completion cDNA can be stored at -20°C until use.

Plasma microRNA Quantification Protocol

Droplet generation and miRNA specific PCR amplification

Materials

- ddPCR Supermix for Probes (no dutp) (Cat #: 186-3024)
- Droplet Generator Oil for Probes (Cat #: 1863005)
- DG8 Cartridges for QX200/QX100 (Cat #: 1864008)
- Drop Generator Holder (Cat #: 1863051)
- Rubber Gasket (Cat #: 1863009)
- PCR Deep Well Plate (Cat #: 12001925)
- Foil Seal (Cat #: 1814040)
- FAM-MGB labeled, target-specific TaqMan Tm Probes (20X) (see Table 1 for catalogue numbers)
- VIC-MGB labeled, miR-39-specific TaqMan Tm Probes (20X) (cel-miR-39-3p, 4427975, Assay ID 000200, Accession # MIMAT0000010)

Procedure

1. Prepare a ddPCR master mix for miR-39 and each microRNA target according to the following table.

Component	Volume per Reaction
ddPCR Supermix (no dutp)	12.5 µL
Target-specific FAM Probe (20X)	1.25 µL
miR-39 VIC Probe (20X)	1.25 µL
**Total**	**15 µL**

2. Vortex and transfer 15 µL into the appropriate reaction tubes.
3. Pipette 5 µL of the target-specific cDNA and 5 µL of the miR-39 cDNA into each reaction. The final volume for the multiplex reactions will be 25 µL.
4. Generate droplets using the DG8 droplet generator following the following the manufacturer's instructions.
5. Transfer **40 µL** of the droplets into the 96 deep-well PCR plate.
6. Seal with foil.
7. Perform PCR using the following thermocycler conditions:

   Thermocycler Conditions: 95°C for 10 minutes (ramp 2°C/sec); 94°C for 30 seconds (ramp 2°C/sec); 60°C for 1 minute (ramp 2°C/sec); repeat at step 2, 39 times; 98°C for 10 minutes (ramp 2°C/sec); hold at 4°C indefinitely; lid temperature: 105°C. Following PCR, the droplets may be stored at 4°C for no more than 24 hours. Do not freeze and proceed to droplet reading and data analysis.

8. Following PCR, incubate the plate at room temperature for 5 minutes before proceeding.
9. Perform droplet reading using the Bio-Rad QX-200 following the manufacturer's instructions.
10. Proceed to Droplet reading and data analysis steps detailed in the associated technical note.
